# The Effectiveness and Practical Application of Different Reduction Techniques in Burst Fractures of the Thoracolumbar Spine

**DOI:** 10.3390/jcm14134700

**Published:** 2025-07-03

**Authors:** Jan Cerny, Jan Soukup, Lucie Loukotova, Marek Zrzavecky, Tomas Novotny

**Affiliations:** 1 Department of Orthopaedics, Faculty of Health Studies, Jan Evangelista Purkyne University in Usti nad Labem and Masaryk Hospital, 401 13 Usti nad Labem, Czech Republic; jan.cerny2@kzcr.eu (J.C.); soukup07@kzcr.eu (J.S.); marek.zrzavecky@kzcr.eu (M.Z.); 2 Department of Orthopaedic Surgery, Faculty of Medicine in Hradec Kralove, Charles University, 500 03 Hradec Kralove, Czech Republic; 3 Department of Rehabilitation and Sports Medicine, Second Faculty of Medicine, Charles University and University Hospital Motol, 150 06 Prague, Czech Republic; 4 Department of Mathematics, Faculty of Science, Jan Evangelista Purkyne University in Usti nad Labem, 400 96 Usti nad Labem, Czech Republic; lucie.loukotova@ujep.cz

**Keywords:** burst fractures, spine trauma, spine reduction, ligamentotaxis, spinal stenosis, spinal canal area

## Abstract

**Background:** The objective was to evaluate and compare the efficacy of direct fragment impaction, indirect reduction through ligamentotaxis, and the combination of both techniques in burst fractures of the thoracolumbar (TL) spine. **Methods:** The fractures were categorized using the Arbeitsgemeinschaft für Osteosynthesefragen (AO) classification and assessed via standard computed tomography (CT) scans for spinal canal area (SCA) and mid-sagittal diameter (MSD). The Frankel classification was used to assess neurological deficits. Only single vertebrae AO types A3 and A4 thoracic or lumbar fractures were included. All patients received bisegmental posterior stabilization, one of the reduction techniques, and, if neurological deficits were present, a spinal decompression. Mean preoperative (µSCApre/µMSDpre), postoperative (µSCApost/µMSDpost) and difference (∆SCA/∆MSD) in radiographic values were obtained and analyzed using the Mumford formula. The significance of the reduction from preoperative stenosis was assessed using a t-test, while the effectiveness of the reduction techniques was compared using the Kruskal–Wallis test and Dunn’s post hoc test. The manuscript was focused primarily on radiographic outcomes; therefore, aside from the neurostatus, no other clinical parameters were statistically analyzed. **Results:** Thirteen patients (38.2%) received stand-alone indirect reduction, 13 patients (38.2%) underwent direct reduction, and a combined reduction was used in eight patients (23.6%). All methods resulted in a statistically significant reduction in spinal canal stenosis (*p* < 0.05), with a minimal mean ∆SCA of 19%. Patients in the direct reduction group had significantly higher µSCApre values compared to those in the indirect reduction group (*p* = 0.02). **Conclusions:** All of the tested reduction techniques provided a significant reduction in spinal canal stenosis. Patients who underwent mere direct reduction had significantly higher preoperative spinal canal stenosis compared to the indirect reduction group.

## 1. Introduction

The treatment of thoracolumbar (TL) spine burst fractures remains a subject of ongoing debate worldwide [1], primarily due to the broad “grey zone” in surgical indications. Some studies favor conservative management, while others advocate for surgical intervention. Surgery generally offers better radiological outcomes, such as preserving vertebral height and reducing post-traumatic kyphosis, but these radiographic improvements do not always translate into superior clinical outcomes [2].

Consensus exists that neurological deficits or suspicion of posterior ligamentous complex (PLC) injury necessitate surgery, as reflected in the Thoracolumbar Injury Classification and Severity Score (TLICS) [2]. However, the specifics of “optimal” surgical procedures remain unclear. According to Magerl’s principles, decompression without instrumentation is insufficient, as it risks neurological deterioration and iatrogenic instability [3]. Today, rigid fixation, either posterior transpedicular or anterior somatic, has become standard, with decompression performed when neurological deficit is present. Additional procedures such as fracture reduction, intersomatic fusion, or anterior column support are tailored individually, often guided by criteria like McCormack’s load-sharing score [4].

Fracture reduction aims to prevent or correct sagittal (kyphosis) and frontal (scoliosis) deformities, as well as to improve fragment positioning within the spinal canal, thereby promoting healing and bony fusion [4]. The commonly used perioperative reduction techniques—direct impaction, ligamentotaxis, or their combination—are limited by factors such as the severity of canal stenosis, bone quality, and ligament integrity [5].

In this study, we aimed to evaluate the efficacy of various reduction techniques, both alone and combined, in surgically treated TL burst fractures. Given the lack of comparative data reflecting Central European experiences, this research addresses a crucial gap. Since the TL region is the most frequently traumatized spinal area, with the highest rate of operatively treated patients [1], our goal was to determine whether the reduction in preoperative canal stenosis was significant and to compare the effectiveness of different techniques. An important point was also to evaluate any differences in preoperative stenosis among the patient groups. This could help us better objectify the decision-making algorithm in choosing a specific technique according to the severity of the stenosis.

## 2. Materials and Methods

### 2.1. Ethical Standards

This cohort study was designed as retrospective, observational and monocentric. It has been conducted in accordance with the ethical standards in the 1964 Declaration of Helsinki and compliance with the approved protocol and Good Clinical Practice. The trial has been reviewed and approved by the Ethics Committee of the Masaryk Hospital in Usti and Labem, Czech Republic. Informed consent to participate in this study and to share medical data was obtained from all patients at the first regular postoperative check-up.

### 2.2. Study Population and Classifications

We retrospectively included 34 patients (24 men and 10 women) with comminuted thoracic or lumbar fractures (AO types A3 and A4) who underwent perioperative fracture reduction between 2020 and 2024. The mean age at surgery was 36.4 years (range 17–73). Potential neurological deficits were assessed using the Frankel classification, from E to A, pre- and postoperatively. Surgical indication was based on key criteria: any initial neurological impairment (Frankel D or higher), confirmed objectively by a neurologist, was an absolute indication. The TLICS classification was not consistently applied, as routine magnetic resonance imaging (MRI) scans were not performed for all patients. PLC injury was usually evaluated indirectly by CT, via interspinous widening, or directly during surgery. Significant destruction of the vertebral body (involving more than 50% of the anterior and middle column height) with possible segmental kyphosis (McCormack score ≥ 6) was also a strong indication for surgery; such cases often warranted a second-stage anterior procedure, as described below.

### 2.3. Surgical Procedures and Hardware

In patients where indirect reduction was performed (either alone or in combination with direct reduction), we always used Ennovate^®®^ instrumentation (B. Braun, Aesculap AG, Tuttlingen, Germany) (Ennovate). In patients where only direct reduction was performed, we additionally implemented the CD Horizon^®®^ Legacy™ (Medtronic Sofamor Danek USA, INC, Memphis, TN, USA) and uCentum™ (Ulrich GmbH & Co. KG, Ulm, Germany) systems.

**Figure 1:**
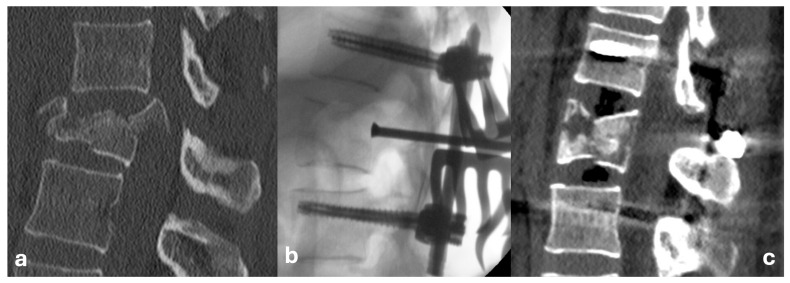
Combined reduction technique: (**a**) CT sagittal plane projection (bone window) of an L1 AO Type A4 fracture; (**b**) Perioperative C-arm guided lateral projection radiograph of a direct fracture reduction using an impactor; (**c**) CT sagittal plane projection showing final lordosis achieved through ligamentotaxis.

The surgical procedure was typically performed within 48 h of trauma for neurologically intact patients, while individuals with neurological deficits underwent surgery in the shortest possible interval, no later than 6 h. The operations were performed in the prone position, through a standard posterior midline approach. Screws were inserted transpedicularly according to Magerl’s technique under radiographic control using a conventional C-arm in two projections. Given the relatively younger cohort of patients and generally good bone quality, a bisegmental instrumentation (2 + 2 screws) was always sufficient. Depending on the individual patient, this procedure was either definitive or, based on the postoperative CT findings, it was decided to perform an additional anterior approach (typically transthoracic partial or complete corpectomy with the placement of an expandable cage, augmented with autologous bone grafts or synthetic bone substitutes). In cases of lower lumbar vertebral injuries (L2 and below), anterior approaches were conducted through lumbotomy. In patients with neurological deficits, decompression was consistently achieved through wide laminectomy. For neurologically intact patients, this procedure was primarily performed when spinal canal stenosis exceeded 50%, as a preventive measure against potential neurological deterioration during postoperative mobilization, and to aid in the direct reduction of the fracture. We performed the direct reduction by “pushing” the dislocated fragments into place at the posterior wall of the injured vertebral body using a dedicated impactor. This technique (alone or in combination) was chosen in cases of spinal canal stenosis estimated at over 50–60%, as assessed by the indicating surgeon, and in all cases of neurological deficits. Indirect reduction was typically performed in patients with segmental deformities, usually with less significant stenosis of the spinal canal (i.e., <50%), and only if there was no necessity for direct exposure of the canal. To achieve indirect reduction, we employed specialized instrumentation to perform ligamentotaxis. This technique involves applying targeted tension to the longitudinal ligaments of the spinal column by applying lever forces to the inserted screws. In certain cases, both direct and indirect reduction techniques were used (Figure 1).

### 2.4. Radiographic Measurements and Software Calculations

Within the selected cohort, we retrospectively measured and subsequently compared preoperative and postoperative values of the spinal canal area (SCA) and its anteroposterior dimension, or the Mid-Sagittal Diameter (MSD). The assumption was that a postoperative CT scan would always be performed. Complete demographic data and basic CT parameters are presented in Table 1.

**Figure 2:**
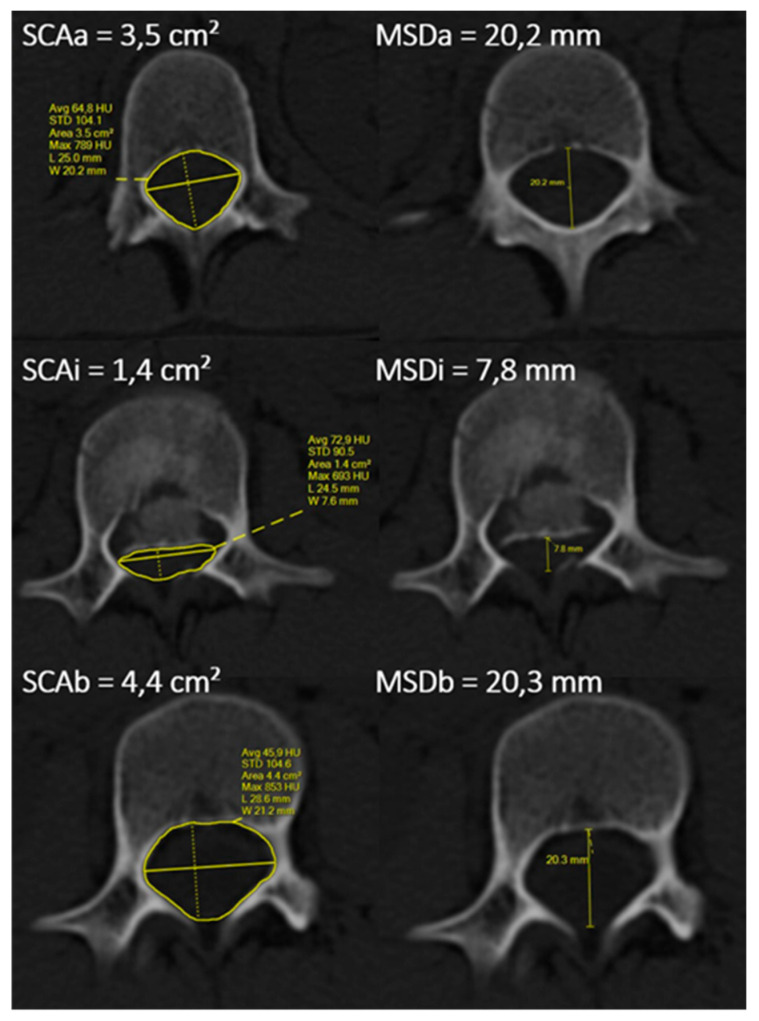
The analysis of SCA (the yellow lines encircling the inner boundaries of the spinal canal) and MSD (the yellow median plane lines) parameters is presented. Standard CT transverse plane projections (bone window) were used. SCAa/MSDa indicates the level (vertebra) above the fracture, SCAi/MSDi describes the fractured vertebra, and SCAb/MSDb designates the level below the fracture.

For the analysis of the values, we utilized the standard “Picture Archiving and Communication System” (Marie PACS, MPACS-5241) software. The SCA value is obtained by delineating the internal cortical lines of the spinal canal at the desired level (using the polygonal region of interest (ROI) function), while the MSD is determined by marking the central (median) plane and measuring the dimension again within the internal cortical lines of the spinal canal. The parameters were then automatically calculated through PACS. Subsequently, we analyzed the reduction of both SCA and MSD in the traumatized level compared to the average values in the segments above and below the fracture (Figure 2). For instance, an SCA of 35% signifies that the spinal canal area has been reduced by 35% due to the fracture.

The formulas used to calculate SCA and MSD were inspired by the foundational work of Mumford and Weinstein [6] and are as follows.

$$SCA\left(stenosis\%\right)=\frac{\frac{SCAa+SCAb}{2}-SCAi}{\frac{SCAa+SCAb}{2}}\times 100\;MSD\left(stenosis\%\right)=\frac{\frac{MSDa+MSDb}{2}-MSDi}{\frac{MSDa+MSDb}{2}}\times 100$$

### 2.5. Postoperative Follow-Up

After surgery, all patients were initially admitted to the orthopedic intensive care unit (ICU), then typically transferred to a regular ward the next day. Patients with persistent neurological deficits were evaluated by a neurologist postoperatively, and a dedicated spinal care protocol was implemented, including positioning, pressure ulcer prevention, neurorehabilitation with respiratory therapy, protein supplementation, bowel and bladder management, vitamin B12 administration, and other interventions. Early communication with the local spinal unit was initiated for patients with severe paraparesis or paraplegia to coordinate ongoing care.

Postoperative CT scans were performed in the ICU to confirm instrumentation placement, evaluate reduction via reanalysis of SCA and MSD, and assess the need for anterior procedures. Starting on postoperative day one, neurologically intact patients began mobilization and ambulation, often using an elastic lumbar brace, a three-point corset (Jewett) or a thoracolumbosacral orthosis (TLSO). Once independent walking was achieved, patients were discharged home or transferred to local rehabilitation facilities.

Follow-up visits took place at 6 weeks post-op, 3 months, 6 months, 12 months, and annually thereafter. At each visit, X-rays were obtained, with occasional CT scans if bony fusion was uncertain. The corset was typically discontinued after 6 weeks if X-rays showed maintained reduction, normal sagittal and frontal balance, and clinical absence of limiting pain. Patients gradually increased activity, but recreational sports or work involving forward bending were generally permitted no earlier than 3 months post-injury.

### 2.6. Statistical Analysis

Basic descriptive statistics were calculated for all variables (mean, minimum, maximum, median, standard deviation). Normality of the variables was assessed visually using Q-Q diagrams and tested with the Shapiro–Wilk test. The assessment of the degree of reduction in preoperative stenosis expressed as ∆SCA and ∆MSD was conducted using a *t*-test. Additionally, 95% confidence intervals were constructed for the mean values of ∆SCA and ∆MSD for the different types of reductions. Due to violations of normality and homoscedasticity in some variables, the Kruskal–Wallis test and additional Dunn’s test with Holm’s correction were utilized to compare the various types of reduction techniques. The significance level was set at *p* < 0.05 for all tests. Statistical analysis was performed using R version 4.4.0 and Microsoft Excel (within Microsoft 365).

## 3. Results

The most frequently operated level was L1 (12 times, 35.4%), followed by Th12 (9 times, 26.5%), L2 (6 times, 17.6%), L4 (3 times, 8.8%), L3 (2 times, 5.9%), and finally Th8 and L5 each once (2 times, 2.9%). There were 24 neurologically intact individuals (Frankel E) (70.7%), while three patients (8.8%) had a preoperative Frankel grade of D, three patients grade C (8.8%), one individual grade B (2.9%), and three patients grade A (8.8%). The development of the neurological status following reduction is depicted in Table 1. Decompression was performed in 17 cases (50%), namely in 11 patients (32.4%) in the direct reduction group and in six patients (17.6%) in the combined reduction group. No decompression was performed in the indirect reduction group. The anterior procedure was indicated in 13 patients (38.2%), namely in four patients each in the indirect and direct reduction groups (11.8% + 11.8%) and five patients in the combined reduction group (14.6%).

The development of the selected radiographic parameter values after fracture reduction is highlighted in Table 2.

**Figure 3:**
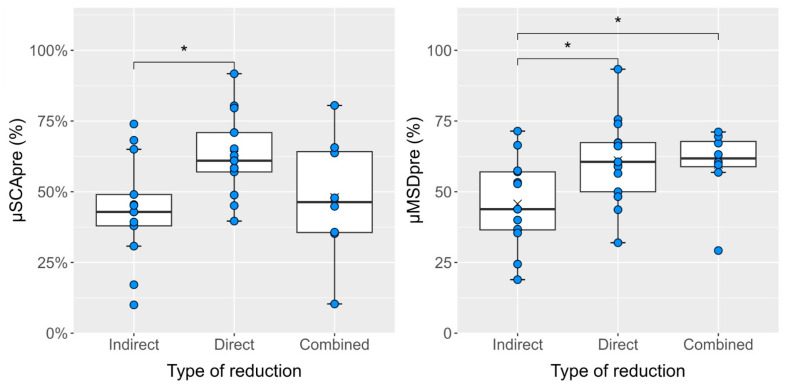
Kruskal–Wallis test and post hoc Dunn test show significant differences in the percentage (%) values of µSCApre and µMSDpre between some groups of patients divided according to the repositioning technique used (* denotes a statistically significant difference with *p* < 0.05). The blue dots indicate the % values of µSCApre and µMSDpre for individual patients.

In the boxplot illustrating the Kruskal–Wallis test results (Figure 3), the average values of ∆SCA and ∆MSD for all three patient groups were marked with a cross.

From a statistical standpoint, it can be stated that all implemented reduction techniques achieved a significant reduction in spinal canal stenosis, with a minimum average value of ∆SCA of 19 p.p. (*p* < 0.05 in all cases). Furthermore, no significant difference was found in the effectiveness of the individual techniques when compared. In the direct reduction group, significantly higher values of µSCApre were found compared to the indirect reduction group (*p* = 0.02; the median µSCApre in the direct reduction group was 61%, while in the indirect reduction group, it was 42.9%). Conversely, in the indirect reduction group, the lowest values of µMSDpre were recorded, with differences compared to the other groups also found to be statistically significant (in both cases, *p* < 0.05; the median µMSDpre in the indirect reduction group was 43.8%, while in the remaining two groups, the median exceeded 60%).

## 4. Discussion

The relationship between surgical and conservative treatment for TL burst fractures remains controversial. Although Wood et al. (2015) [7] reported lower Oswestry Disability Index and VAS scores in non-operated patients at long-term follow-up (16–20 years post-injury), no study conclusively shows better outcomes with conservative management. Most authors agree on the superiority of surgical treatment based on improved radiological results, but these do not always translate into better clinical outcomes [8]. Our future research should therefore incorporate objective clinical data to better correlate radiographic findings with patient-centered outcomes.

The 2021 analysis by the World Federation of Neurological Societies [4] established criteria for surgical therapy of burst fractures, including 25–30 degrees of traumatic segmental kyphosis, loss of vertebral body height greater than 50%, and spinal canal stenosis of more than 50%. However, optimal methods for surgical management based on these criteria were not clearly defined. Based on our experience, we typically treat most burst fractures surgically, assuming the patient’s overall condition permits it. We believe this approach offers significant advantages, including the potential for faster rehabilitation and a reduced incidence of post-traumatic deformities, which can be challenging to correct at a later stage. However, it is important to note that this belief is not yet supported by firm data.

Huang et al. (2020) [5] published a similar analysis of 60 patients who underwent perioperative reduction of TL spine fractures. Specifically, in 33 individuals, a combination of direct and indirect reduction techniques was implemented, and in 27 cases, only indirect reduction was performed. In the combined reduction group, the ∆SCA or the decrease in “encroachment ratio” of 25.5% ± 4.3% was achieved, while in the indirect reduction-only group, it was 21.5% ± 1.4%. The study resulted in a significant difference in the effectiveness of the reduction, favoring the combined technique. A limitation of this study was the difference in the technique of indirect reduction compared to our work, as it was performed solely by manual pressure on the spinous processes in the intervened segment, followed by tightening the fixator in the corrected position, thus without the use of specialized instrumentation.

The limitations of “stand-alone” ligamentotaxis are well known. For example, in fractures with a positive “reverse cortical sign”—a 180° rotation of a displaced bony fragment into the spinal canal separated from the vertebral body—indirect reduction is contraindicated due to the risk of paradoxical fragment dislocation and potential dural or neural injury [9]. Additionally, below L3, the residual posterior longitudinal ligament (PLL) fibers are typically too sparse to enable effective reduction. However, a case has been reported at L3 with over 90% preoperative stenosis, where ligamentotaxis nearly normalized the SCA, reducing stenosis to only 10% [10]. In our study, the lowest preoperative SCA (µSCApre) was observed in the ligamentotaxis-only group, suggesting this technique was mainly used in neurologically intact patients with less than 50% stenosis. This pattern is also noted in the work by Benek et al. (2021) [9], but there remains a lack of studies objectively evaluating this instinctive decision-making process to develop a clear treatment algorithm.

Regarding indirect reduction, the necessity of MRI to assess PLL integrity is debatable. MRI is traditionally considered the “gold standard,” with up to 78% specificity for the PLL [11] and about 90% sensitivity for detecting interspinous ligament injuries, a key component of the PLC [12]. However, CT-based skeletal parameters, such as the vertebral body compression ratio, also provide reliable, indirect information about the central spinal column and PLL integrity [11]. Practical considerations, like MRI availability and time requirements, can delay surgical management. Nevertheless, MRI remains essential for patients with neurological deficits not explained by bone trauma (SCIWORA), to rule out conditions such as disc herniation or epidural hematomas [13].

An important aspect that must be considered when assessing the significance of stenosis is the level of the fracture. In the original Hashimoto analysis [14], it was concluded that patients with burst fractures at the levels of Th11 or Th12 have a significant risk of developing neurological deficits already at a spinal canal stenosis of 35% or more. At the level of L1, this increases to 45% or more, and at levels L2 and below, it is 55% and more. Similarly, in the study by Yüksel et al. (2016) [15], it was noted that a critical spinal canal stenosis at the TL junction (specific segmental distribution was not provided) is 40%, with 81.6% of patients with this level of stenosis or greater exhibiting some degree of neurological deficit.

Fracture reduction in patients with severe osteoporosis presents unique challenges. The brittle bone tissue increases the risk of “cutting through” during reduction or screw pull-out due to insufficient fixation. To mitigate these risks, various strategies, such as multi-segment stabilization, bicortical screw fixation, and cement-augmented or expandable screws, have been recommended [16]. Principles also differ for osteoporotic patients with predominantly compressive fractures; therefore, the Deutsche Gesellschaft für Orthopädie und Unfallchirurgie (DGOU) classification is preferred over the traditional AO system, as it guides options like vertebroplasty and kyphoplasty. However, these techniques carry potential complications like cement leakage and embolism [17]. Despite technological advances, aggressive attempts at anatomical reduction are generally discouraged in osteoporotic cases [18]. Future developments in CT imaging are expected to improve osteoporosis detection during routine scans without additional tests [19]. In our study, osteoporosis was not routinely assessed, partly due to the younger average age of our cohort.

Prior to modern instrumentation (mainly in the 1970s–1990s), preoperative reduction, often via 24–48 h of spinal realignment in Böhler suspension, was common for burst fractures. Based on radiographs, patients were then operated on or fitted with a plaster corset in the corrected position [20]. Today, this method is rarely used, likely due to advances in surgical options and accessibility. In our institution, we employ preoperative realignment for patients who are not operated on immediately, such as those with comminuted fractures involving up to about 50% spinal canal stenosis or with traumatic segmental kyphosis.

Finally, we have mentioned that the decision for a specific reduction technique was partially based on the surgeon’s decision and preference. Several studies have investigated the influence of biases and the availability of second opinions (SOs) on spine surgery decision-making. This factor is especially relevant in elective spine surgical care. For instance, Gattas et al. [21] found that SOs can differ from the original treatment plan in more than 75% of cases. Moreover, numerous subjective and personal factors that can negatively impact the final surgical outcome have been identified by Little et al. [22] These include, e.g., overconfidence, framing (misleading interpretation of possible complications), confirmation (seeking advice among “likely-to-agree” colleagues), etc. To our knowledge, no study has specifically focused on surgeon decision-making biases in the treatment of TL spine burst fractures. However, we are fully aware that this factor could negatively impact the generalizability of our outcomes. Therefore, we have made every effort to minimize its impact and to operate in accordance with evidence-based practices and recommendations.

We believe our study can represent a notable advancement for surgeons managing these injuries daily. Simple radiographic measurements based on widely available CT scans can expedite decision-making regarding the optimal surgical approach. With future, more clinically focused research on this topic, we hope to at least partially address the ongoing controversy surrounding the preferred treatment strategy.

### Limitations and Bias

A limitation of our study was the exclusive focus on radiographic outcomes of surgical therapy, without corresponding correlations to clinical findings. Additionally, we encountered limitations in software capabilities when measuring SCA and MSD, particularly due to the difficulty in precisely delineating the posterior cortical line of the spinal canal after laminectomy. Another factor was the possible impact of the surgeon’s bias as mentioned above.

## 5. Conclusions

To our knowledge, this is the first Central European study to provide reproducible outcomes in the management of spinal canal stenosis after TL trauma, mainly due to standardized surgical techniques and easy-to-follow radiographic measurements. All reduction techniques provided a significant reduction in preoperative SCS. Regarding the ∆SCA and ∆MSD parameters, there were no significant interindividual differences between the patient groups. Mean µSCApre in the direct reduction patient group was significantly higher compared to the other groups, which could favor this method in cases of severe (>50%) spinal canal stenosis.

## Figures and Tables

**Table 1:** Demographic data and radiographic parameters of the study population. M = male; F = female; y = years; AO = Arbeitsgemeinschaft für Osteosynthesefragen; SCApre = Spinal Canal Area preoperative; MSDpre = Mid-Sagittal Diameter preoperative; preOp = preoperatively; postOp = postoperatively.

Patient	Fracture (AO)	Reduction	Neurostatus (preOp→postOp)	SCApre	MSDpre
M, 17y	L2 A4	Combined	E→E	35.2%	60.4%
F, 51y	L1 A4	Combined	E→E	35.7%	59.5%
F, 31y	L2 A3	Direct	E→E	48.8%	50%
M, 63y	L1 A4	Indirect	E→E	49%	57%
F, 73y	L4 A4	Direct	E→E	57%	75.6%
M, 22y	L4 A4	Direct	D→E	39.6%	48.3%
M, 23y	L1 A4	Combined	E→E	63.7%	67.2%
M, 24y	L4 A3	Indirect	E→E	45.5%	43.8%
F, 24y	T12 A4	Combined	A→A	44.8%	56.8%
M, 30y	L1 A3	Indirect	E→E	17.1%	18.9%
M, 34y	L1 A3	Indirect	E→E	10%	24.4%
F, 30y	L2 A4	Combined	D→E	65.6%	63.2%
F, 32y	L1 A4	Indirect	E→E	68.2%	66.4%
M, 31y	L1 A4	Direct	C→D	80.4%	67.4%
M, 31y	L2 A3	Indirect	E→E	37.9%	36.5%
F, 27y	L3 A3	Direct	E→E	79.6%	74%
M, 29y	L1 A4	Combined	E→E	80.5%	69.5%
M, 29y	L5 A4	Direct	D→E	58.4%	43.7%
M, 32y	L2 A4	Indirect	E→E	74%	57.4%
F, 33y	L1 A3	Direct	E→E	65.2%	67.3%
M, 26y	T12 A3	Indirect	E→E	42.9%	36.8%
M, 34y	T12 A4	Indirect	E→E	42.9%	53.3%
F, 26y	T12 A4	Direct	E→E	45.1%	32%
M, 46y	L1 A4	Direct	E→E	70.9%	58.9%
M, 73y	T12 A4	Indirect	C→D	39.3%	52.8%
M, 37y	T12 A4	Direct	A→C	62.8%	66.2%
M, 40y	T8 A4	Combined	A→A	10.3%	29.2%
M, 43y	T12 A4	Indirect	E→E	30.8%	40%
M, 44y	L1 A4	Indirect	E→E	65%	71.4%
M, 44y	L2 A4	Direct	C→E	91.7%	93.3%
F, 63y	L1 A3	Indirect	E→E	45.2%	35.4%
M, 22y	L3 A4	Combined	E→E	47.8%	71.1%
F, 41y	T12 A4	Direct	B→C	58.3%	56.5%
M, 34y	T12 A4	Direct	E→E	61%	60.56%

**Table 2:** The effectiveness of different reduction techniques based on selected radiographic parameters. No. = number; ∆SCA = difference/change (preoperative to postoperative) in spinal canal area value; ∆MSD = difference/change (preoperative to postoperative) inf mid–sagittal area value; p.p. = percentage points.

Method	No. of Patients	µSCApre	µSCApost	∆SCA	µMSDpre	µMSDpost	∆MSD
Indirect	13 (38.2%)	43.7%	16.6%	27.1 p.p.	45.7%	20.8%	24.9 p.p.
Direct	13 (38.2%)	63%	12.7%	50.3 p.p.	61%	29.7%	31.3 p.p.
Combined	8 (23.6%)	48%	11.6%	36.4 p.p.	59.6%	26.1%	33.5 p.p.

## Data Availability

The data presented in this study are available on request from the corresponding author due to the necessity of maintaining the anonymity of the included patients.
